# Nævus verruqueux géant chez une adolescente de 15 ans: à propos d’un cas

**DOI:** 10.11604/pamj.2018.31.50.11408

**Published:** 2018-09-24

**Authors:** Békaye Traoré, Lamissa Cissé

**Affiliations:** 1 CNAM (ex Institut Marchoux), Bamako, Mali

**Keywords:** ævus, géants, verruqueux, Blaschko, Nevus, giant, verrucous, Blaschko

## Abstract

Verrucous nevus is a benign tumor showing a linear pattern on Blaschko lines. It is caused by mosaic mutations of the receptor FGFR3 gene. It manifests as an aesthetic disfigurement, but individuals even experience functional complications due to itchiness. Lesions may be localized or extended (giant). In our context, the scarcity of specialized centers in dermatology is a cause of diagnostic delay inducing the patient to undertake unsuitable therapies responsible for infectious or degenerative complications. We report the case of a 15-year old girl with a history of keratotic papules showing a linear pattern along the neck, the right upper limb, the flank since childhood. Histological examination confirmed the diagnosis of verrucous nevus.

## Image en médecine

Le nævus verruqueux est une tumeur bénigne à disposition linéaire suivant les lignes de Blaschko. Cette affection est due à des mutations en mosaïque du gène du récepteur FGFR3. Elle représente surtout un préjudice esthétique, mais aussi fonctionnel en raison des manifestations prurigineuses, les lésions peuvent être localisées ou étendues (géantes). Dans notre contexte, la rareté des centres spécialisés en dermatologie constitue un facteur de retard diagnostique incitant le patient à entreprendre des thérapeutiques peu appropriées, responsables de complications infectieuses ou dégénératives. Nous rapportons le cas d’une adolescente de 15 ans qui présente depuis l’enfance des lésions papuleuses keratosiques disposées en bande de façon linéaire le long du cou, du membre supérieur droit, du flanc. L’examen histologique confirme le diagnostic de nævus verruqueux.

**Figure 1:**
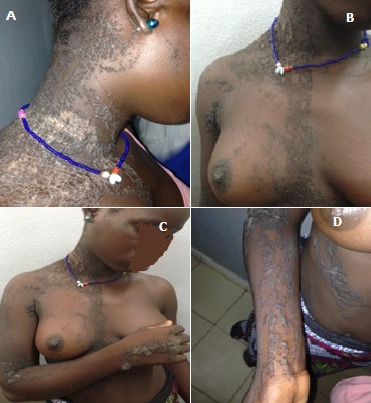
A) Lésions verruqueuses linéaires face latérale droite du cou; B) lésions linéaires mediothoracique (vue de face); C) gros plan, buste et membre supérieur droit; D) flanc droit, plis du coude et avant-bras droit (extension)

